# *Cornus mas* and *Cornus officinalis*—A Comparison of Antioxidant and Immunomodulatory Activities of Standardized Fruit Extracts in Human Neutrophils and Caco-2 Models

**DOI:** 10.3390/plants10112347

**Published:** 2021-10-30

**Authors:** Monika E. Czerwińska, Agata Bobińska, Katarzyna Cichocka, Tina Buchholz, Konrad Woliński, Matthias F. Melzig

**Affiliations:** 1 Department of Biochemistry and Pharmacogenomics, Faculty of Pharmacy, Medical University of Warsaw, Banacha 1, 02-097 Warsaw, Poland; 2 Centre for Preclinical Research, Medical University of Warsaw, Banacha 1B, 02-097 Warsaw, Poland; 3 Student Scientific Association “Farmakon”, Department of Biochemistry and Pharmacogenomics, Medical University of Warsaw, Banacha 1, 02-097 Warsaw, Poland; agata.bobinska2997@gmail.com (A.B.); katarzyna.cichocka.kc@gmail.com (K.C.); 4 Institute of Pharmacy, Freie Universitaet Berlin, Königin-Luise-Str. 2+4, D-14195 Berlin, Germany; tina.buchhol@gmail.com (T.B.); matthias.melzig@fu-berlin.de (M.F.M.); 5 Polish Academy of Sciences Botanical Garden, Center for Biological Diversity Conservation in Powsin, Prawdziwka 2, 02-973 Warsaw, Poland; k.wolinski@obpan.pl

**Keywords:** loganin, loganic acid, dogwood, inflammation, traditional medicine

## Abstract

Fruits of *Cornus mas* and *Cornus officinalis* are representative plant materials traditionally used in Europe and Asia, respectively, in the treatment of diabetes and diabetes-related complications, which are often mediated by pathogenic inflammatory agents. Additionally, due to the fact of mutual infiltration of Asian and European medicines, the differentiation as well as standardization of traditional prescriptions seem to be crucial for ensuring the quality of traditional products. The objective of this study was a comparison of biological activity of extracts from fruits of *C. mas* and *C. officinalis* by an assessment of their effect on reactive oxygen species (ROS) generation in human neutrophils as well as cytokines secretion both in neutrophils (tumor necrosis factor *α*, TNF- *α*; interleukin 8, IL-8; interleukin 1*β*, IL-1*β*) and in human colon adenocarcinoma cell line Caco-2 (IL-8). To evaluate the phytochemical differences between the studied extracts as well as to provide a method for standardization procedures, a quantitative analysis of iridoids, such as loganin, sweroside, and loganic acid, found in extracts of *Cornus* fruits was performed with HPLC-DAD. All standardized extracts significantly inhibited ROS production, whereas the aqueous-alcoholic extracts were particularly active inhibitors of IL-8 secretion by neutrophils. The aqueous-methanolic extract of *C. officinalis* fruit, decreased IL-8 secretion by neutrophils to 54.64 ± 7.67%, 49.68 ± 6.55%, 50.29 ± 5.87% at concentrations of 5, 50, and 100 µg/mL, respectively, compared to LPS-stimulated control (100%). The aqueous extract of *C. officinalis* fruit significantly inhibited TNF-*α* release by neutrophils at concentrations of 50 and 100 µg/mL. On the other hand, the aqueous-ethanolic extract of *C. mas* fruit showed the propensity to increase TNF-*α* and IL-1*β* secretion. The modulatory activity of the *Cornus* extracts was noted in the case of secretion of IL-8 in Caco-2 cells. The effect was comparable with dexamethasone. The content of loganin in aqueous and aqueous-methanolic extract of *C. officinalis* fruit was higher than in the aqueous-ethanolic extract of *C. mas* fruit, which was characterized by a significant quantity of loganic acid. In conclusion, the immunomodulatory effect observed in vitro may partially confirm the traditional use of *Cornus* fruits through alleviation of the development of diabetes-derived inflammatory complications. Loganin and loganic acid are significant markers for standardization of *C. mas* and *C. officinalis* fruit extracts, respectively.

## 1. Introduction

Herbal products have been used traditionally for health purposes all over the world [1]. In particular, traditional Chinese medicine (TCM), which originated 2500 years ago, is widely used in Asia but is more and more often prevalent all over the world, in particular in Europe. The traditional medicine of China is a health care system characterized by a unique philosophical concept of therapies and diagnostic practices. In addition to unconventional practices, TCM include herbal therapies and dietary supplementation [2]. It is more and more often supported by conventional medicine because its effectiveness is still debated. The evidence on TCM is controversial and commonly criticized due to the methodological properties of scientific reports or their biased dissemination caused by language limits [2]. Nevertheless, the concept of relationship between food and medicine, which is proposed by TCM, guarantees body homeostasis and provide health benefits both for preventing and treating diseases. It stays in agreement of Hippocrates’s statement “Let thy food be thy medicine and medicine be thy food” [3]. Last but not least, the identity and quantity of plant materials or their preparations, which are the components of plant-derived medicinal products, are necessary for ensuring the safety and efficacy of these herbal products. The complex composition of plant extracts and herbal medicine products is determined by a wide range of factors such as production process, the extraction solvent, physical state of the plant material, and last but not least the drug extract ratio (DER). That all needs to be stated to guarantee identity of plant materials or plant-derived preparations as well as facilitate comparison of herbal preparations [4]. However, it seems that the market of plant-derived products both in Europe and Asia has been heterogenous due to botanical and ethnopharmacological misleadings found in herbal products. Therefore, the efforts of regulatory environments are focused on the herbal medicinal market in Europe and Asia, and its harmonization as well as regulation of medicinal products, food and other related to them such as nutraceuticals [1].

*Cornus mas* L. and *Cornus officinalis* Sieb. et Zucc. (*C. officinalis* Torr. ex Dur.) are European and Asian, respectively, representatives of dogwoods belonging to Cornaceae [5]. Despite *C. mas* known as cornelian cherry can be found in southwest Asia, it is mainly widespread in southern and central Europe [6]. It is used both as a traditional medicine and even more often in food industry for production of liquors, jams, jellies, and fruit drinks [7,8]. On the other hand, *C. officinalis* (Japanese cornel, Asiatic dogwood) is native to China, Korea, and Japan, where it is considered as one of 25 vegetable drugs and prescriptions such as Liuwei Dihuang (China), Paeng-Jo-Yeon-Nyeon-Baek-Ja-In-Hwan (Korea), as well as Ba-Wei-Di-Huang-Wan (Japan). In the Chinese culture it is named “Shanzhuyu”. In addition, fruit of *C. officinalis* was included in the Chinese Pharmacopoeia [9,10]. The prescriptions containing this plant material have been particularly used for the treatment of renal diseases, revitalization of kidneys and liver, diabetes, chronic inflammation as well as to stimulate brain function, improve memory, and prolong the lifespan [9]. The indications for use of plant materials from *C. mas* include gastrointestinal disorders, diarrhea, stomach ulcers, inflammatory bowel disease and urinary tract infections, sore throats as well as liver and kidney diseases [6].

Most available literature concerns anti-diabetic properties of *Cornus* fruit preparations. A wide range of reports showed the effect of extracts from fruits of *C. officinalis* on diabetes development [11,12,13,14] as well as diabetes-derived complications such as nephropathy [15,16,17] and neurodegeneration [18]. In addition, compounds which were found in fruit of *C. officinalis* such as morroniside, loganin, and ursolic acid ameliorated diabetes-associated damages and complications, including promoting glucose uptake, inhibiting *α*-glucosidase activity, and oxidative stress as well as down-regulation of inducible nitric oxide synthase (iNOS), nuclear factor-κB (NF-κB), and cyclooxygenase-2 (COX-2) [19,20,21]. One of the polyphenols of *Corni fructus*, 7-*O*-galloyl-D-sedoheptulose, down-regulated the levels of advanced glycation end products (AGE)-related proteins as well as some inflammation-related protein expressions in hepatic tissues [22]. In addition, a lot of research was focused on antioxidant activity of extracts from fruits of *C. mas* [23,24,25,26] and *C. officinalis* [27]. However, there are no data comparing the activity of the extracts from these two species in vitro.

The role of inflammation has been widely considered in pathophysiology of diabetes mellitus and metabolic disorders recently. Targeting the inflammatory pathways, including ROS and cytokines which are implicated in the inflammation of pancreatic *β*-cells, in order to improve prevention and diabetes control seems to be a challenge and perspective for future diabetes therapies. On the other hand, gut microbiota has been widely discussed to be also implicated in the pathophysiology of diabetes. In particular, microbe-derived product such as lipopolysaccharide (LPS) is believed to affect immune cells, induce secretion of cytokines, and lead to low-grade inflammation, which is specific molecular background for development of obesity and diabetes [28]. In recent studies, it was shown that phytochemicals of *C. officinalis* fruit, such as loganin and morroniside, had alleviated dextran sodium sulfate-induced colitis in mice [29]. We hypothesize that apart from gut-microbiota-derived products, the developing systemic inflammation may also impair the intestinal barrier or at least delay the resolution of inflammation in the gut through unlimited chemotaxis of leukocytes. Therefore, the resolution of inflammation both in the innate immune system and in the intestinal epithelium seems to be a good strategy to support the control of diabetes development in addition to conventional anti-diabetic therapy lowering blood glucose. Taking into consideration that extracts from fruits of *C. mas* and *C. officinalis* inhibited digestive enzymes or glucose uptake [30,31], their anti-inflammatory potential would support their anti-diabetic properties used in the traditional therapies.

Both *C. mas* and *C. officinalis* belongs to a subgenus, which is characterized by blooming before leaf development and having red or purple black fruits [32]. Taking into consideration the similar morphological characters of *Cornus* fruits, it seems crucial to focus on their differentiation based on the phytochemical profile. To date, the compounds from classes of compounds such as iridoids, anthocyanins, flavonoids, and tannins were particularly identified in extracts from fruits of *C. mas* [7,8,33,34] and *C. officinalis* [35,36,37]. In particular, morroniside and its derivatives along with loganin are noted as the major iridoids found in fruits of *C. officinalis* [36], whereas loganic acid and cornuside are more often detected in fruits of *C. mas* [7]. In addition, dogwood fruits are listed as a source of vitamin C and organic acids [38,39].

Due to the fact of mutual infiltration of Asian and European medicines, particularly from China to the West of Europe, the differentiation as well as standardization of traditional prescriptions seem to be crucial for ensuring the quality of traditional products. Therefore, we decided to develop a universal method for standardization of preparations from plant materials such as fruits of *C. mas* (CM) and *C. officinalis* (CO), which are traditionally used in Europe and Asia, respectively. The goal of this study was also a comparison of antioxidant activity of *Cornus* extracts through inhibition of oxidative burst in human neutrophils (PMNs). Bearing in mind the increasing interest in low-grade inflammation and its role in the development of a wide range of inflammation-related disorders, we purposed to track the cytokines secretion in PMNs (TNF-*α*, IL-8, IL-1*β*) as well as in human colon adenocarcinoma cell line Caco-2 (IL-8). According to our knowledge this is the first report providing the data on the activity of standardized CM and CO extracts affecting cytokines pathways in *ex vivo*-derived immune cells and in vitro intestinal epithelial cells.

## 2. Results

### 2.1. ROS Generation in PMNs

**Figure 1:**
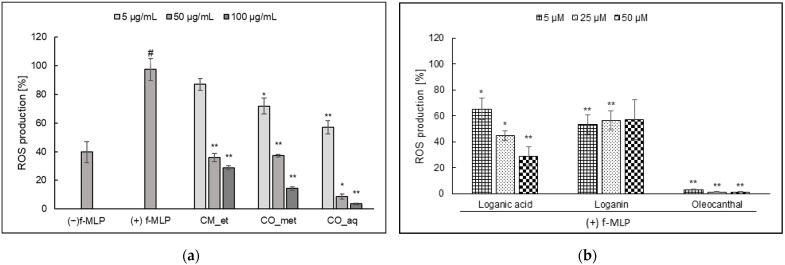
f-MLP-stimulated ROS production by PMNs (mean ± SEM [%]) after the treatment by: (**a**) *Cornus* extracts; (**b**) standard compounds. CM_et—aqueous ethanolic extract of *Cornus mas* fruit, CO_met—aqueous-methanolic extract of *Cornus officinalis* fruit, CO_aq—aqueous extract of *Cornus officinalis* fruit; ^#^ *p* < 0.001 vs. (−) LPS; * *p* < 0.05, ** *p* < 0.001 vs. (+) LPS.

The *Cornus* fruit extracts inhibited ROS generation induced by formyl-met-leu-phenylalanine (f-MLP) in PMNs. It was established that the higher concentration of extracts the lower ROS production (Figure 1a). The percentage of ROS was 28.86 ± 1.40, 14.36 ± 1.26, and 3.49 ± 0.68 (*p* < 0.001) for aqueous-ethanolic extract of CM (CM_et), aqueous-methanolic extract of CO (CO_met), and aqueous extract of CO (CO_aq) at concentration of 100 µg/mL, respectively, in comparison to f-MLP stimulated control cells (97.39 ± 7.81%). In addition, we studied the inhibition of ROS production by loganic acid (1) and loganin (2). It was established that loganin (2) did not show the concentration-dependent activity, whereas loganic acid (1) inhibited ROS generation (*p* < 0.05) in the concentration-dependent manner (Figure 1b). However, none of *Cornus* iridoids exerted as significant antioxidant activity as oleocanthal (secoiridoid) used as positive control (*p* < 0.001).

### 2.2. Cytokines Secretion by PMNs

#### 2.2.1. IL-8

**Figure 2:**
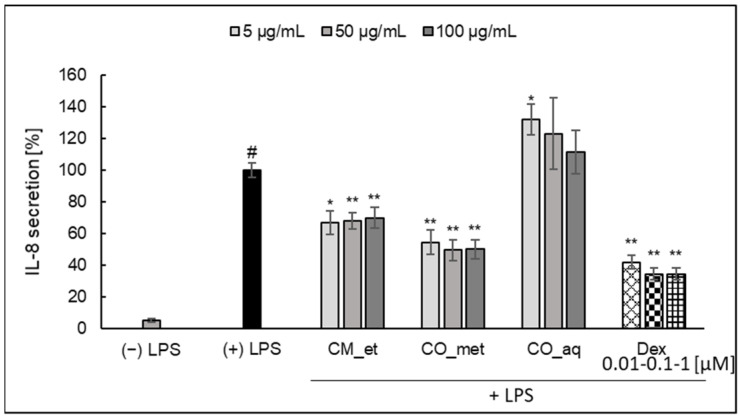
Effect of *Cornus* extracts on LPS-stimulated IL-8 secretion by PMNs (mean ± SEM [%]). CM_et—aqueous ethanolic extract of *Cornus mas* fruit, CO_met—aqueous-methanolic extract of *Cornus officinalis* fruit, CO_aq–aqueous extract of *Cornus officinalis* fruit, Dex—dexamethasone; ^#^ *p* < 0.001 vs. (−) LPS; * *p* < 0.05, ** *p* < 0.001 vs. (+) LPS.

Both CM_et and CO_met extracts significantly inhibited IL-8 secretion by PMNs (Figure 2). The activity of aqueous-alcoholic extracts (*p* < 0.001) was only slightly weaker than the activity of known glucocorticosteroid, dexamethasone (*p* < 0.001). However, the effect was not concentration-dependent in the tested concentration range. On the other hand, the aqueous extract of *C. officinalis* caused the increase of IL-8 secretion (at concentration of 5 µg/mL, *p* < 0.05).

#### 2.2.2. TNF-*α*

**Figure 3:**
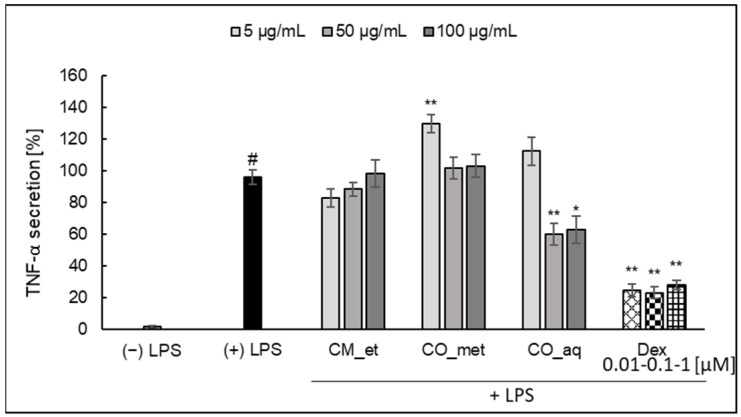
Effect of *Cornus* extracts on LPS-stimulated TNF-α secretion by PMNs (mean ± SEM [%]). CM_et—aqueous ethanolic extract of *Cornus mas* fruit, CO_met—aqueous-methanolic extract of *Cornus officinalis* fruit, CO_aq—aqueous extract of *Cornus officinalis* fruit, Dex—dexamethasone; ^#^ *p* < 0.001 vs. (−) LPS; * *p* < 0.05, ** *p* < 0.001 vs. (+) LPS.

The extract of *C. mas* fruit did not affect TNF-*α* release by PMNs (Figure 3). The most significant inhibitory effect was detected in the case of CO_aq extract at concentration of 50 µg/mL (*p* < 0.001) and 100 µg/mL (*p* < 0.05). In the case of this cytokine CO_met at the lowest concentration of 5 µg/mL significantly increased TNF-*α* (*p* < 0.001). The activity of CO_met extract at concentrations of 50 and 100 µg/mL was not significant as in the case of CM_et extract (*p* > 0.05).

#### 2.2.3. IL-1*β*

**Figure 4:**
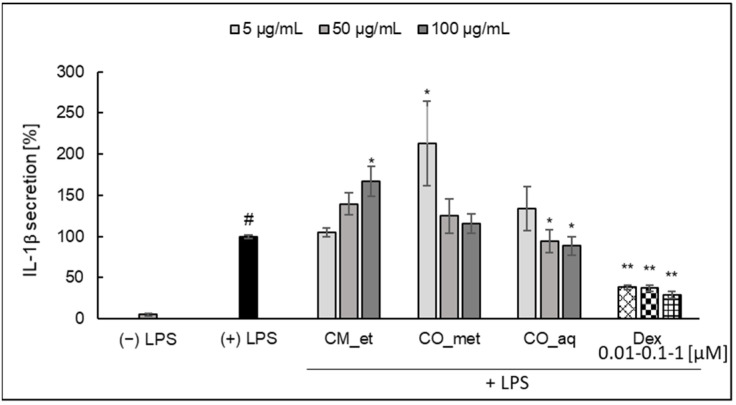
Effect of *Cornus* extracts on LPS-stimulated IL-1*β* secretion by PMNs (mean ± SEM [%]). CM_et—aqueous ethanolic extract of *Cornus mas* fruit, CO_met—aqueous-methanolic extract of *Cornus officinalis* fruit, CO_aq—aqueous extract of *Cornus officinalis* fruit, Dex—dexamethasone; ^#^ *p* < 0.001 vs. (−) LPS; * *p* < 0.05, ** *p* < 0.001 vs. (+) LPS.

The increase of IL-1*β* secretion by PMNs treated with increasing concentrations of CM_et extract (at concentration of 100 µg/mL*, p* < 0.05) was observed (Figure 4). On the other hand, when the cells were incubated with *C. officinalis* extracts at concentrations of 50 and 100 µg/mL, PMNs secreted less IL-1*β* than in the case of treatment with extracts in the concentration of 5 µg/mL.

### 2.3. IL-8 Secretion in Caco-2 Cell Line

**Figure 5:**
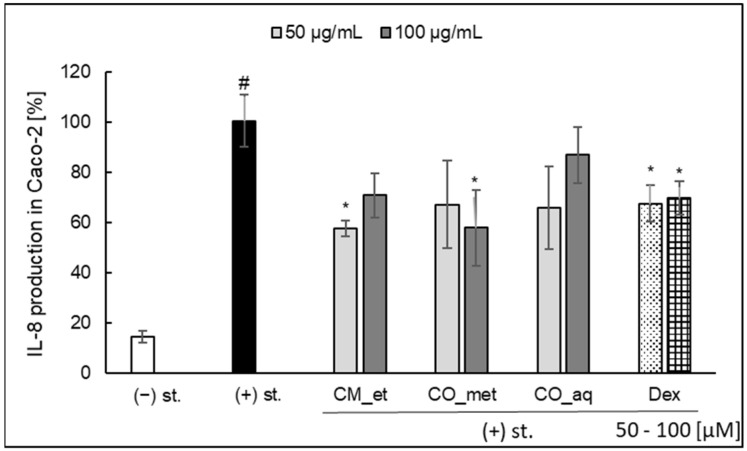
Effect of *Cornus* extracts on IL-8 secretion by Caco-2 (mean ± SEM [%]). CM_et—aqueous ethanolic extract of *Cornus mas* fruit, CO_met—aqueous-methanolic extract of *Cornus officinalis* fruit, CO_aq—aqueous extract of *Cornus officinalis* fruit, Dex—dexamethasone, st.—mixture of IL-1*β*/TNF-*α*/IFN-*γ*/LPS; ^#^ *p* < 0.001 vs. (−) LPS; * *p* < 0.05 vs. (+) LPS.

In our preliminary study in Caco-2 cell model, we observed the modulatory activity of the *Cornus* extracts in the case of secretion of IL-8 in Caco-2 cells (Figure 5). Only CM_et (50 µg/mL; 57.58 ± 3.29%; *p* < 0.05) and CO_met (100 µg/mL; 57.83 ± 15.06%; *p* < 0.05) significantly inhibited IL-8 release by Caco-2 cells when compared with stimulated control (100.46 ± 10.21%). It should be underlined that the activity of extracts was quite similar to the effect of dexamethasone used as a positive control. It is worth to note that the mixture of IL-1*β*, TNF-*α*, interferon *γ* (IFN-*γ*), and LPS was used to stimulate the epithelial cells for IL-8 production.

### 2.4. PMNs and Caco-2 Cells Viability after Cornus Extracts Treatment

**Figure 6:**
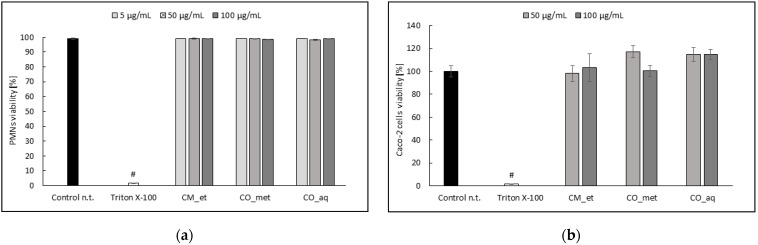
Effect of *Cornus* extracts on cells viability (**a**) PMNs; (**b**) Caco-2 cells. Data are expressed mean ± SEM [%]. Control n.t.—non-treated control cells; ^#^ *p* < 0.001 vs. control n.t.

The extracts did not affect significantly (*p* > 0.05) the cells viability at the concentrations used in the model assays (Figure 6).

### 2.5. Qualitative Analysis of Cornus Extracts

#### 2.5.1. Results of Validation Procedures

The parameters such as selectivity, linearity, repeatability, recovery as well as limit of detection (LOD) and limit of quantification (LOQ) were considered in order to validate the HPLC-DAD method developed for quantification of iridoids in extracts from fruits of *C. mas* and *C. officinalis*. The results of validation procedures were presented in Table 1. The retention times for standard compounds were as follows: 19.679 min for loganic acid (1), 23.782 min for loganin (2), and 24.043 min for sweroside (3). The chromatograms of standards confirming the purity and selectivity of compounds were provided in Supplementary Materials (Figures S1–S3). The repeatability of loganic acid (1) was 168.61 ± 0.06 µg (CV: 0.33%), 32.39 ± 0.05 µg (CV: 1.45%), and 34.67± 0.06 µg (CV: 1.81%) %) in CM_et, CO_met, and CO_aq, respectively. The repeatability of loganin (2) was 112.30 ± 0.03 µg (CV: 0.31%) and 106.43 ± 0.18 µg (CV: 1.66%) in CO_met and CO_aq, respectively. The repeatability of sweroside (3) was 6.65 ± 0.01 µg (CV: 2.05%), 17.92 ± 0.03 µg (CV: 1.87%), and 17.17 ± 0.02 µg (CV: 0.95%) in CM_et, CO_met, and CO_aq, respectively. Recovery of loganic acid was determined at the level 100.29 ± 8.33% (100% of content) and 100.28 ± 0.94% (120% of content). In the case of loganin in CO_aq the recovery ranged from 99.99% (100% of content) to 102.98% (120% of content).

#### 2.5.2. Quantification of Iridoids in Cornus Extracts

**Figure 7:**
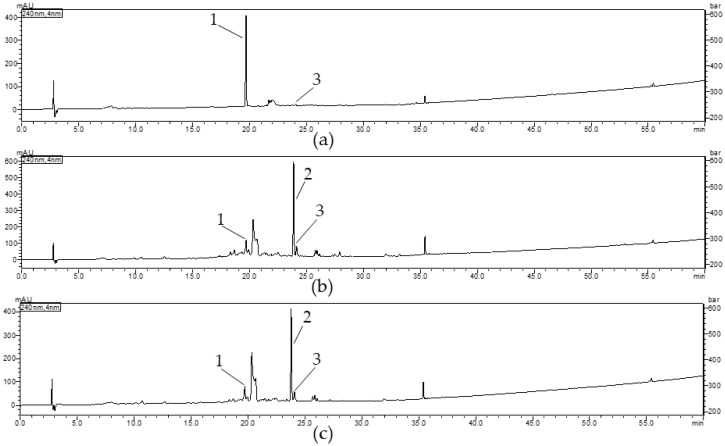
HPLC chromatograms registered at 240 nm (**a**) aqueous-ethanolic extract of *Cornus mas* fruit; (**b**) aqueous-methanolic extract of *Cornus officinalis* fruit; (**c**) aqueous extract of *Cornus officinalis* fruit. Loganic acid (1), loganin (2), sweroside (3).

Loganic acid (1) was the most abundant compound of CM_et, whereas the peak of loganin (2) dominated both in CO_met and CO_aq. In addition, another iridoid such as cornuside (not described in Figure 7) was registered in all tested extracts at Rt = 35.5 min. However, due to the limited quantity of this compound, it was not taken into consideration in this study.

The results of quantitative analysis of iridoids in *Cornus* fruit extracts was shown in Table 2. In particular, the significant contents of loganic acid (1) in Cm_et and loganin (2) in both CO extracts were noted.

## 3. Discussion

Inflammation is considered as a response to local injury of cells, which is mostly resolved by an increase of blood flow and leukocytes infiltration, as well as increase production of a host cellular mediators released against toxic agents. The repair of the damaged tissues allows resumption of their balanced functions [40,41]. The monolayer of intestinal epithelium is considered both a first physical and immunological barrier that protects against gut microbiota. When the epithelium is injured, PMNs after recruitment to the site of infection activate their defense mechanism, such as ROS and lytic enzymes production or release of neutrophil extracellular traps, against invading pathogens. Moreover, production of matrix metalloproteases as well as secretion and modification of cytokines or chemokines in PMNs may affect inflammatory conditions through recruitment of additional effector cells [42]. The severity of the bowel diseases and colitis was established to correlate with PMNs infiltration [42]. When the mononuclear cells are absent, the deleterious functions of PMNs are rather revealed [41].

The resolution of inflammation seems to be crucial for termination of pro-inflammatory pathways [40]. The imbalance between pro- and anti-inflammatory host systems is the main risk factor for progression of a chronic low-grade inflammation, which is considered more and more often as a feature of metabolic syndrome, diabetes mellitus type 2 or cardiovascular diseases [40]. The dual role of PMNs, either beneficial for resolution of pathogen-derived inflammation or deleterious for tissue damage, has been considered. Both functional deficiency and hyperactivity of PMNs may lead to intestinal inflammation, tissue damage, and in consequence to system diseases, including diabetes complications. Therefore, the functions of PMNs are critical for maintenance of intestinal homeostasis [42]. On the other hand, it is highlighted that the activity of PMNs due to their sensitivity to chemoattractant gradient strictly depends on other immune cells during colitis [41].

Therefore, the constituents of plant medicines may affect both directly the intestinal epithelium or indirectly immune cells by their bioavailable metabolites. The functionality of both intestinal and immune cells may be strictly interdependent in particular in the case of mucosal tissue damage. Lipopolysaccharide, which is an endotoxin found in the membrane of Gram-negative bacteria, enters the host system and promotes the progression of inflammation in the case of bacteria rupture and intestinal wall leakage [43,44]. It is known that LPS acts by stimulating innate immune signaling via Toll-like receptor, which induces translocation of NF-κB and transcription of TNF-*α*. The host cells can modulate the pathogen inflammatory response by modifying microbial molecules such as LPS. It is worth to note that other factors than gut microbiota, including alkaline phosphatase, play a crucial role in gut immune system. In general, the intestinal epithelial cells, similar to our sodium butyrate-differentiated Caco-2 cell model, secrete an isoform of alkaline phosphatase, which is able to dephosphorylate the LPS endotoxin [45]. In addition, intestinal alkaline phosphatase mediates in the recruitment of PMNs to intestinal lumen in response to TNF-*α* [45]. Both TNF-*α* and IL-1*β* can induce intestinal epithelial cells for production of a wide range of pro-inflammatory cytokines, including IL-8 (CXCL8), which is a potent neutrophil chemoattractant [46]. However, the secretion of this cytokine by human intestinal epithelial cells in vitro depends on cell line. In addition, the degradation of LPS by alkaline phosphatase in differentiated intestinal cells causes that LPS itself could not have been used for Caco-2 cells stimulation. In our study, we established the experimental conditions for IL-8 secretion by Caco-2 cell line based on the available literature. In general, the combinations of TNF-*α* with IFN-*γ*, IL-6 or epidermal growth factor were established to show positive and synergistic effect on IL-8 secretion in different human intestinal epithelial cell lines. It was previously shown that Caco-2 cells had secreted IL-8 in response to IL-1*β* stimulation in contrary to stimulation with TNF-*α* and LPS [47]. For this reason, in order to stimulate Caco-2 cells for secretion of IL-8 we used the mixture of IL-1*β*, TNF-*α*, IFN-*γ*, and LPS [48]. This combination of different physiological agonists let us to track the possible effects of plant constituents on interdependent pathways of intestinal cells and leukocytes. In addition, we hypothesized that transmural inflammation, epithelial ulceration, fissure formation, which are characteristic for intestinal disorder, may be a gate for plant-derived molecules that may affect and modulate the immune system. For this reason, in order to explain the role of plant extracts in the resolution of inflammation related to chronic diseases it is justified to study the effect of plant extracts on leukocytes functions along with their effect on intestinal epithelium cells.

The secretion of IL-8 was significantly inhibited both in PMNs and Caco-2 cells, in particular by aqueous-alcoholic extracts from *Cornus* fruits. In fact, we did not observe the concentration-dependent activity of extracts. However, the significant difference in the activity of extracts at different concentrations was not noted in the previous studies. According to previous reports the extract of CM containing 15 and 30 mg of total phenols/b.w./day reduced IL-1*β* after 2 h, 24 h, and 48 h from induction of inflammation with carrageenan in paw oedema model in rats. In the meantime, TNF-*α* secretion was decreased after 2 h but it was the same as the control group after 24 h and 48 h. The differences of activity exerted by extracts at both doses were not highlighted [49]. It is worth to note that our study is the first report on effectiveness of *Cornus* extracts on IL-8 level, which is significant observation due to the chemoattractive role of this cytokine.

Our results show that the fruit extracts at the lowest concentrations may cause the increase of IL-1*β* secretion. The plant-derived class of compounds which is known to increase the cytokines secretion are polysaccharides. Few reports provided the data on isolation and structural characterization of polysaccharides found in CO [50,51,52] or CM [53]. It was previously established that polysaccharide fractions isolated from fruits of *Lycium barbarum* (LBP), which is also used in the prescriptions of TCM, enhanced the production of ROS, TNF-α, IL-6, and NO as well as the phagocytosis of RAW 264.7 macrophages [54]. However, it was also suggested that LBP as an immunostimulator may enhance an immune response or prevent immune damage caused by excessive activation of macrophages [55]. Therefore, we hypothesized that in *Cornus* extracts at the lower concentrations polysaccharides may play more significant role in the stimulation of cells for cytokine secretion than other compounds. For this reason, an increasing secretion is observed. On the other hand, iridoids quantified in the *Cornus* fruit extracts may affect and modulate the cytokines secretion when the extract is applied at the higher concentrations. In particular, loganin (2) found in a high concentration in CO extracts may play such role.According to the available literature CO extracts are a source of a wide range of iridoid compounds whereas loganic acid (1) and cornuside are mainly described in CM extracts [5]. Other compounds, such as ursolic acid, morroniside or cornuside described in CO extracts, might exert synergistic or additive effect due to their described suppressive activity against inflammatory mediators [56,57,58]. It was recently established that loganin at the doses of 80 and 160 mg/kg and morroniside at the doses of 90 and 180 mg/kg significantly decreased the expression of IL-1*β* and TNF-α in colon tissues of mice with dextran sodium sulfate-induced colitis [29]. The contrary effect, such as increasing secretion of IL-1*β* in particular, was observed in the case of CM extract rich in loganic acid (1) in our study. It is worth to note that cornuside, which inhibitory activity against IL-1*β* or TNF-α production in RAW 264.7 cell line was proved, was also detected in CM extract but it seems that its concentration is too low in comparison to loganic acid (1) to influence the cytokines secretion.

It is considered that IL-1*β* is particularly important for stimulation of Caco-2 cells [48]. Therefore, the overproduction of IL-1*β* or TNF-*α* in PMNs might lead to stimulation of epithelial cells for production of IL-8 and further development of inflammation in consequence. However, the reduction of chemoattractant such as IL-8 both in PMNs and Caco-2 after the treatment of *Cornus* extracts in a direct mechanism or at least via inhibition of ROS production provides the immunomodulatory effect of tested in this study extracts.

Furthermore, it is worth to note that we established the significant antioxidant activity of extracts. All tested preparations inhibited ROS generation in PMNs in a concentration-dependent manner. Additionally, the data on the direct antioxidant activity of loganic acid (1) and loganin (2) are rather limited. In our comparative study we established that loganic acid (1) inhibited ROS production in PMNs in a concentration-dependent manner whereas the activity of loganin (2) was stable in a concentration range 5–100 µg/mL. It is supposed the methylation of carboxylic moiety found in the structure of loganin (2) limits its antioxidant activity in comparison to loganic acid (1). Recent report by Dzydzan et al. showed that loganic acid decreased intracellular ROS content and restored the antioxidant defense system balance by increasing activities of catalase (85%), glutathione peroxidase (24%) as well as the content of reduced glutathione (21%) in leukocytes of streptozotocin-induced diabetic rats [59].

The correlations between PMNs count, inflammatory markers, such as IL-8, TNF-*α*, IL-6, as well as the unprompted, f-MLP or LPS-stimulated ROS production were described in the literature [60]. In particular, PMNs count, and spontaneous ROS production positively correlated with TNF-*α* in the elderly participants of the study, whereas the correlation between IL-8, ROS production as well as the PMNs count was established in young participants [60]. During a systemic inflammatory response ROS are signaling molecules, which mediate the modulation of crucial events such as phagocytosis, gene expression, secretion of pro-inflammatory mediators, and apoptosis. The result of this activity is dysregulation of inflammation [61]. In physiological conditions, NADPH oxidase system is responsible for endogenous ROS production. This system regulates tyrosine phosphorylation dependent pathways, which further modulate the expression of cytokines and chemokines via NF-κB within inflammatory response. In addition, the membrane permeability for ROS may influence intracellular signaling pathways in adjacent cells placed in the inflammatory site. Therefore, under pathological conditions similar to the case of tissue injury, excess production of ROS by immune cells may destroy endothelial or epithelial cells [60]. For this reason, searching the intercellular correlations and effect of antioxidants-rich plant extracts on inflammation-based disorders, we decided to perform our study with two populations of cells engaged in the intestinal inflammation. In general, Ogawa et al. concluded that the unprompted and increasing ROS production from PMNs seems to be age-dependent and therefore it is highly possible that it is connected with prevalent age-associated immune dysregulation [60]. Therefore, the antioxidant-rich therapy by restoration of metabolic balance of endogenous radicals might be a way for promotion of circulation, nourishing blood, and removing blood stasis, which are the purposes of TCM [62].

According to our knowledge the antioxidant activity of *Cornus* extracts or fresh fruits was studied in vitro. The antioxidant activity of fresh cornelian cherries was comparable in ABTS (677.88 µmol Trolox equivalents/100 g FW) and FRAP (628.75 µmol Trolox equivalents/100 g FW) assays [49]. Similarly, the aqueous-ethanolic extract from fruits of CO in the concentration range from 10 to 100 µg/mL exerted antioxidant potential in ABTS and FRAP assays in addition to scavenging of DPPH radical and inhibition of LPS-induced ROS generation in RAW 264.7 macrophages. Moreover, the extract of CO fruit increased the expression of superoxide dismutase isoforms, catalase as well as glutathione peroxidase [63].

A comparison of quantity of iridoids in extracts prepared from fruits of CM and CO have never been performed so far. The content of loganic acid (1) in fruit of CM ranged from 81.5 to 461.1 mg/100 g fresh weight according to previous report [7]. On the other hand, the data on presence of loganin (2) in CM fruits are rather limited and uncertain. For this reason, we rather confirmed that loganin (2) did not occur in fruits of CM. We suppose that loganic acid (1) is more stable iridoid form in CM fruit due to the presence of organic acids and acidity of the plant material [64]. On the other hand, the content of loganin (2) in extracts from CO fruit in different maturation stages ranged from 13.3 µg/mg extract to 18.0 µg/mg [65]. Our results stay in agreement with that data.

Considering severe diabetes-related complications, hyperglycemia in particular changes in the internal balance of body fluids, including hypertonicity, extracellular solute, osmotic diuresis, and water intake, leads to nephropathy. For this reason, controlling hydration as well as management of fluid and electrolyte administration constitute significant challenges in patients with hyperglycemia, in particular this dialysis-related [66]. The balanced supply of water and electrolytes in addition to dietary sugars restrictions should be taken into account when fruit extracts are considered in diabetes therapy. As far as *Cornus* fruit extracts, in particular CM, concerned they might be components of functional beverages. Nevertheless, apart from phytochemicals quantities the content of carbohydrates, electrolytes as well as defined osmolality should be designed for functional recovery drink used for supporting diabetes therapy [67].

The main concept of TCM is promotion of vital energy (named “qi”). The remedies are chosen based on the symptoms and signs which are usually known as “qi” deficiency. The diagnosis of “qi” deficiency in TCM does not usually correspond to the Western diagnosis, which includes disorders such as hypertension, diabetes or colitis [2]. Therefore, the scientific data on CO usually provide the results of a wide range of its activities, which do not target the molecular goals, which are specific for these disorders. In addition, some studies are not directly related to the traditional use. Even though they do not confirm the traditional use, they provide some hints on the other biochemical or pharmacological effects as well as indicate the new areas of application [6]. On the other hand, several pharmacological studies of CM were reported for a direct justification of its ethnomedicinal use [68,69,70]. Our study is the first dedicated comparative investigation of CM and CO fruit preparations providing the differences in their phytochemistry and biological activity. We believe that it can provide a support for traditional use of these plant materials in the inflammation-related metabolic disorders.

## 4. Materials and Methods

Acetonitrile HiPerSolv Chromanorm^®^ (20060.320) was purchased from VWR Chemicals (Radnor, PA, USA). HPLC grade methanol (106009) was purchased from Merck (Darmstadt, Germany). Dimethyl sulfoxide (DMSO, 113635509) was purchased from Chempur (Piekary Śląskie, Poland). Water purification system Millipore Simfilter Simplicity UV (Molsheim, France) was used to obtain ultra pure water. Citric acid (CHEM-115382101), sodium citrate tribasic dihydrate (C7254), glucose (114595600) for citrate dextrose solution (ACD) were purchased from Chempur (Piekary Śląskie, Poland). Dextran from *Leuconostoc mesenteroides* (31398-500 G), propidium iodide (PI, P4170-10 MG), dexamethasone (Dex, D1756-25 MG), luminol (09253-1 G), formyl-met-leu-phenylalanine (F3506-10 MG), sodium butyrate (303410-100 G), 4-methylumbelliferyl phopsphate (M8883-100 MG), Triton X-100 (T8787-100 ML), loganin (36483-10 MG-F), and sweroside (SMB00083-1 MG) were purchased from Sigma-Aldrich (Saint Louis, MO, USA). RPMI 1640 medium (R7509-500 ML), amphotericin B (A2942-100 ML), and 3-(4,5-dimethylthiazol-2-yl)-2,5-diphenyltetrazolium (MTT, 0793-5 G) were purchased from Sigma-Aldrich (Saint Louis, MO, USA). Pancoll Human (P04-601000) was from PAN-Biotech (Aidenbach, Germany). Phosphate buffered saline (PBS, L0615-500) and Dulbecco’s modified eagle medium (DMEM, L0103-500) enriched with 3.7 g/L NaHCO_3_ and 4.5 g/L glucose, with stable L-glutamine, low endotoxin, fetal bovine serum (FBS, S1860-500) as well as penicillin-streptomycin (L0022-100) were purchased from Biowest (Nauillé, France). Hanks’ balanced salt solution (HBSS, 14175095) and TrypLE™ Express Enzyme (12604021) were purchased from Gibco Thermo Fisher (Waltham, MA, USA). Recombinant human IL-1*β* protein (IL038), LPS (2552117) from *Escherichia coli* (0111:B4), and formic acid (100264) were obtained from Merck (Darmstadt, Germany). Tris-HCl buffer was prepared with 100 mM Tris (Promega Corporation, Madison, WI, USA) and 300 mM HCl (POCh, Gliwice, Poland). Recombinant tumor necrosis factor-*α* (300-01A) as well as recombinant inteferone-*γ* (300-02) were purchased from PeproTech (Cranbury, NJ, USA). Sets of immunosorbent-assay for human IL-8 (555244), TNF-*α* (555212), and IL-1*β* (557953) were purchased from BD Biosciences (Erembodegem, Belgium). Pierce BCA Protein Assay Kit (23225) were obtained from Thermo Fisher Scientific (Rockford, IL, USA).

The Caco-2 cell line originated from the DSMZ German Collection of Microorganisms and Cell Cultures, Leibnitz Institute (Braunschweig, Germany).

Loganic acid (1) was isolated from the aqueous-ethanolic extract of *C. mas* with preparative HPLC according to the conditions as follows: the stationary phase was Kintex XB-C_18_ column (150 × 21.2 mm, 5 μm; Phenomenex, Torrance, CA, USA); the mobile phase (A) was 0.1% formic acid in water (*v*/*v*), and the mobile phase (B) was 0.1% formic acid in acetonitrile (*v*/*v*); gradient 0–60 min, 5–20% B; elution 20 mL/min; injection 400 µL, and identified as previously described [30].

### 4.1. Plant Materials and Extracts Preparations

Fruits of *Cornus mas* were collected in September 2017 in the Botanical Garden–Center for Biological Diversity Conservation in Powsin (Polish Academy of Sciences, Poland) (52°06′17″ N, 21°05′42″ E). A voucher specimen (No FW25_20160914_CM) of aerial parts from *C. mas* is deposited in the Department of Pharmacognosy and Molecular Basis of Phytotherapy (Medical University of Warsaw, Poland). The plant material was identified by M. E. C. and K. W. according to the guidebook [71].

Preparation of the aqueous-ethanolic extracts from fruits of *C. mas*: A 200-g portion of powdered plant material was macerated three times with aqueous ethanol (60%, *v*/*v*) in a ratio of 1:10 (*m*/*v*) for 24 h each time. The collected aqueous-ethanolic extracts were concentrated under reduced pressure and lyophilized (Telstar Cryodos 50, Terrassa, Spain).

The aqueous and aqueous-methanolic extracts from fruits of. *C. officinalis* were prepared as described previously [72]. The extracts were tested in the concentrations of 5, 50, and 100 µg/mL.

### 4.2. General Experimental Conditions in PMNs Culture

Neutrophils were isolated from buffy coats, which were obtained from healthy volunteers (<35 years old) from the Warsaw Blood Donation Centre (Poland). The isolation procedures were preceded by mixing buffy coat with anticoagulant citrate dextrose solution (ACD). Neutrophils were isolated by dextran sedimentation and centrifugation in density gradient with buffer for leukocytes isolation (Pancoll Human, 1.077 g/mL) according to the Böyum’s method previously described [73]. During isolation procedures residues of erythrocytes were removed by hemolysis with water. Following isolation, the cells were suspended in an appropriate medium, such as RPMI 1640 or HBSS, and were maintained at 4 °C before use [74]. The cells were seeded in 96-well plates for ROS production assays (3.5 × 10^5^ cells/well in HBSS) and for cytokines secretion assays (2 × 10^6^ cells/well in RPMI 1640 medium with 10% FBS, 2 mM L-glutamine, and 10 mM HEPES).

Neutrophils cytotoxicity was assessed by flow cytometry as decribed previously [74]. The cells were treated with extracts at concentrations of 5, 50, and 100 µg/mL for 24 h. Next, they were washed twice with PBS and suspended in 500 µL of PI solution (0.5 μg/mL). The cells were analyzed by flow cytometry FACSCalibur (BD Biosciences, San Jose, CA, USA), and data from 10,000 events were recorded. Cells that displayed high permeability to PI were expressed as a percentage of PI (+) cells. The results of the viability were acquired according to an equation: 100% − PI (+) cells%. TritonX-100 was used as positive control.

The ROS production by f-MLP-stimulated PMNs was determined using chemiluminescence assay as described previously [75]. Cell suspension was mixed with 50 µL of extracts in the concentration range from 5 to 100 µg/mL and 50 µL of luminol (2.5 mM). The ROS production was initiated with 30 µL of f-MLP (1.5 µg/mL). Changes of chemiluminescence were measured over 40 min in the intervals of 2 min in a microplate reader (Synergy 4, Biotek, Winooski, VT, USA). None of extracts itself affected the chemiluminescence produced by stimulated cells. The percentage of inhibition was calculated in comparison to the control without tested extract in the maximum of luminescence. Oleocanthal was used as the positive control.

The cytokine secretion by LPS-stimulated PMNs was determined with enzyme-linked immunosorbent assays (ELISA). The cells were treated with extracts at concentrations of 5, 50, and 100 µg/mL (50 µL) 30 min before the stimulation with 10 µL of LPS (100 ng/mL). Neutrophils were further cultured for 24 h at 37 °C with 5% CO_2_. The control cells were treated with LPS or cultured without LPS [74]. The ELISA assays for released IL-8, TNF-*α*, and IL-1*β* into cell supernatants were performed by following the indications of the manufacturer. The effect on IL-8, TNF-α, and IL-1*β* production was calculated as the percentage of released cytokine in comparison with stimulated control without tested extract (+LPS). Dexamethasone (Dex) was used as the positive control.

### 4.3. General Experimental Conditions of Caco-2 Cell Culture

The Caco-2 cells were cultured in 75 mL cell culture flasks at a seeding density 10^5^ cells/mL in DMEM supplemented with 20% FBS (*v*/*v*), l-alanyl-l-glutamine (2 mM), penicillin (100 IU/mL), streptomycin (100 µg/mL), and amphotericin B (2.5 ng/mL). The cells were passaged when 80% confluency was achieved. They were detached with TrypLE™ Express Enzyme after double washing with PBS. The medium was changed three times a week. The culture was conducted in standard conditions in humidified atmosphere with 5% CO_2_ at 37 °C. The cells in passages range between 20 and 45 were used for all experiments.

The cells were seeded in 24-well plates at a density of 2 × 10^5^ cells/well in 20% FBS medium. After 24 h the medium was changed to DMEM supplemented with 5% FBS (*v*/*v*), l-alanyl-l-glutamine (2 mM), penicillin (100 IU/mL), streptomycin (100 µg/mL), amphotericin B (2.5 ng/mL), and sodium butyrate (5 mM) in order to induce differentiation of adenocarcinoma cells [76], which was conducted for 72 h in final. The Caco-2 cell differentiation was assessed routinely with fluorescent assay based on the activity of alkaline phosphatase as a specific brush border enzyme marker [77]. The enzyme’s activity was evaluated in a cell lysate related to cellular protein according to a modified method previously described [78]. The fluorescence of 4-methylumbelliferone (λ_exc_ = 360 nm, λ_em_ = 465 nm), which is a product of 4-methylumbelliferyl phosphate hydrolysis, was measured at 37 °C in a microplate reader (SYNERGY 4, Biotek, Winooski, VT, USA).

The differentiated Caco-2 cells were cultured overnight in the absence or presence of extracts at concentrations of 5, 50, and 100 µg/mL. Then, the cells were stimulated with the mixture of IL-1*β* (25 ng/mL), TNF-*α* (10 ng/mL), IFN-*γ* (10 ng/mL), and LPS (100 ng/mL) for IL-8 secretion. The cocktail for stimulation was modified based on the available literature [48]. After the treatment by 24 h the supernatants were collected. The released into supernatants IL-8 was measured by ELISA following the indications of the manufacturer. The concentration of detected IL-8 was calculated related to cellular protein content determined spectrophotometrically by the Bradford’s method (BCA Protein Assay Kit). The effect on IL-8 secretion was normalized to control by expression as percentage of released cytokine/mg protein in comparison with stimulated control without tested extract. Dexamethasone (Dex) was used as the positive control.

The differentiated Caco-2 cells cytotoxicity was assessed with MTT assay modified according to the method previously described [79]. The fruit extracts at concentrations of 50 and 100 µg/mL were dissolved in a medium and incubated for 48 h. The positive control was 10% solution of Triton X-100. After treatment cells were washed twice with 500 µL of PBS and incubated with MTT solution (0.5 mg/mL) for 1 h. The reagent solution was replaced with DMSO, and absorbance was measured at 560 nm (test) and 620 nm (reference) in the microplate reader (Synergy 4, Biotek, Winooski, VT, USA).

### 4.4. Chromatography Analysis

The quantitative analysis by HPLC-DAD was performed using Shimadzu HPLC system (Shimadzu, Kyoto, Japan) equipped with a pump LC-10AT, a sampler SIL-20A, and a diode array detector SPD-M20A, a CTO-10AS column oven. The chromatographic separation was conducted on a reversed-phase Kinetex XB-C_18_ (250 × 4.6 mm, 5 μm) column (Phenomenex, Torrance, CA, USA) at 25 °C. A mobile phase consisted of the mobile phase (A) 0.1% formic acid in water (*v*/*v*), and the mobile phase (B) 0.1% formic acid in acetonitrile (*v*/*v*). A multistep gradient solvent system: 0–10 min, 0–15% B; 10–15 min, 5–10% B; 15–20 min, 10–15% B; 20–30 min, 15–2% B, and 30–60 min, 20–95% B was used. The flow rate was 1 mL/min. The column was equilibrated for 10 min between injections (10 µL). UV-vis spectra were recorded over a range of 200–450 nm. The chromatograms for quantification were acquired at 240 nm, 280 nm, and 325 nm. A LabSolutions system (Shimadzu, Kyoto, Japan) was used for operating procedures, calculation, and complete quantitative data.

Accurately weighted amounts (1 mg) of standards of loganic acid (1), loganin (2), and sweroside (3) were dissolved in appropriate volume of 0.1 % (*v*/*v*) formic acid in water (1:1, *v*/*v*) to obtain concentrations 1 mg/mL. The stock solutions of the compounds were further diluted in the same solvent to obtain standard solutions containing: 0.05, 0.1, 0.2, 0.5 and 1 mg/mL for loganic acid (1), and loganin (2) 1 0.05, 0.1, 0.15, 0.3, and 0.5 mg/mL for sweroside (3). Based on the peak areas versus the amount of standards calibration curves for each compound were plotted. The regression parameters of the calibration curves were used to calculate the quantity of compounds.

### 4.5. Method Validation

The method developed was validated, including specificity, linearity, range, accuracy, precision, limit of detection (LOD), and limit of quantification (LOQ), according to the International Conference on Harmonisation (ICH) guidelines [80].

The assignment of the peaks on the chromatograms allowed to evaluate specificity of the method. Retention times, peaks purity and UV spectra of substances in extracts were compared with reference substances.

The peak areas for standards at 5 concentrations in minimum triplicate were assigned. The linear relationship was evidenced by analysis of residual sum of squares (F test).

The recovery was assessed using three concentration levels such as 80, 100, and 120% of the known added amounts of loganic acid (1) and loganin (2) in the sample. Sweroside (3) was not included due to the limited amount of the compound. An extract solution (10 mg/mL) and standard solution (1 mg/mL) in the determined amount (loganic acid: 84.4 µL, 105.5 µL, and 126.6 µL; loganin: 13.58 µL, 16.97 µL, and 20.36 µL) were mixed to obtain appropriate content of the compound in the final volume 10 mL.

The repeatability of six independent sample solutions (intraday) was established for precision determination. The intermediate precision was analyzed in three independent sample solutions on different days (interday).

Limits of detection and quantification were established based on the standard deviation of the response (*σ*–standard deviation of the intercept) and the slope (*S*) of the calibration curves using the formulas: LOD = 3.3 × *σ*/*S* and LOQ = 10 × *σ*/*S*, respectively [80].

### 4.6. Statistical Analysis

The results were displayed as a means ± standard error of mean (S.E.M). All samples were studied in three independent experiments in triplicate. Statistical significance of the differences between means was evaluated by testing a homogeneity of variance and a normality of distribution followed by one-way ANOVA. The non-parametric methods such as Kruskal–Wallis or U-Mann–Whitney’s tests were used if necessary. *p* values below 0.05 were considered statistically significant. The results were analyzed using Excel (Microsoft Polska, Warsaw, Poland) and Statistica 13 (StatSoft, Cracow, Poland) software.

## 5. Conclusions

According to our knowledge, in this study we have compared the activity of standardized extracts from fruits of *C. mas* and *C. officinalis* for the first time. Loganic acid and loganin were indicated as markers for standardization procedures and differentiation of these two plants-derived products. However, we suggest other iridoids such as morroniside or cornuside would be included in the quality control procedures of *Cornus* extracts. The extracts inhibited particularly the secretion of the significant chemoattractant such as IL-8 both by human neutrophils and intestinal epithelial cells of Caco-2 cell line. In fact, the activity of extracts toward IL-8 secretion was different in comparison to the activity against IL-1*β* and TNF-*α*. The influence of other compounds, such as other iridoids or fruit polysaccharides, is taken into consideration. Nevertheless, the immunomodulatory effect of *Cornus* extracts on immune and epithelial cells may provide the host homeostasis and justify the traditional use of these preparations in traditional European and Chinese medicine.

## Figures and Tables

**Table 1:** Method validation parameters for quantified compounds.

Compound	Regression Equation	r	R^2^	Test F	LOQ [µg/mL]	LOD [µg/mL]
Loganic acid (1)	y = 1.6791 × 10^7^ x − 2.0096 × 10^5^	0.9999	0.9997	42,771.41	45.92	15.15
Loganin (2)	y = 1.6791 × 10^7^ x − 2.0096 × 10^5^	0.9999	0.9997	46,182.75	62.59	20.65
Sweroside (3)	y = 1.4243 × 10^7^ x − 50,927.8208	0.9852	0.9707	364.37	17.06	5.63

Test F: *α* = 0.95.

**Table 2:** Contents of the tested compounds in extracts of *Cornus mas* and *Cornus officinalis* fruits [mg/g d.w. of extract] ± SD.

Extract	Loganic Acid (1)	Loganin (2)	Sweroside (3)
CM_et	16.97 ± 0.10	n.d.	0.65 ± 0.02
CO_met	3.27 ± 0.04	11.38 ± 0.08	1.77 ± 0.03
CO_aq	3.40 ± 0.06	10.55 ± 0.13	1.72 ± 0.02

n.d.—not detected.

## Supplementary Materials

The following are available online at https://www.mdpi.com/article/10.3390/plants10112347/s1, Figure S1: UV chromatogram of loganic acid (1) registered with HPLC-DAD at λ = 240 nm, Figure S2: UV chromatogram of loganin (2) registered with HPLC-DAD at λ = 240 nm, Figure S3: UV chromatogram of sweroside (3) registered with HPLC-DAD at λ = 240 nm, Figure S4: The chemical structures of loganic acid (1), loganin (2), and sweroside (3).
