# Supplementary material for: Cornus mas and Cornus officinalis—A Comparison of Antioxidant and Immunomodulatory Activities of Standardized Fruit Extracts in Human Neutrophils and Caco-2 Models
Source: Plants (Basel). 2021 Oct 30;10(11):2347. doi: 10.3390/plants10112347 (PMC8618406; doi:10.3390/plants10112347)
Supplement: Supplementary file 1 [file plants-10-02347-s001.zip › plants-1421657-supplementary.pdf]

plants

Supplementary Materials

Cornus mas and *Cornus officinalis*—A Comparison of Antioxidant and Immunomodulatory Activities of Standardized Fruit Extracts in Human Neutrophils and Caco-2 Models

Monika E. Czerwińska [1,2,*], Agata Bobińska [3], Katarzyna Cichocka [3], Tina Buchholz [4], Konrad Woliński [5] and Matthias F. Melzig [4]

[1] Department of Biochemistry and Pharmacogenomics, Faculty of Pharmacy, Medical University of Warsaw, Banacha 1, 02-097 Warsaw, Poland
[2] Centre for Preclinical Research, Medical University of Warsaw, Banacha 1B, 02-097 Warsaw, Poland
[3] Student Scientific Association "Farmakon", Department of Biochemistry and Pharmacogenomics, Medical University of Warsaw, Banacha 1, 02-097 Warsaw, Poland; agata.bobinska2997@gmail.com (A.B.); katarzyna.cichocka.kc@gmail.com (K.C.)
[4] Institute of Pharmacy, Freie Universitaet Berlin, Königin-Luise-Str. 2+4, D-14195 Berlin, Germany; tina.buchhol@gmail.com (T.B.); matthias.melzig@fu-berlin.de (M.F.M.)
[5] Polish Academy of Sciences Botanical Garden, Center for Biological Diversity Conservation in Powsin, Prawdziwka 2, 02-973 Warsaw, Poland; k.wolinski@obpan.pl
* Correspondence: monika.czerwinska@wum.edu.pl; Tel.: +48-221-166-185

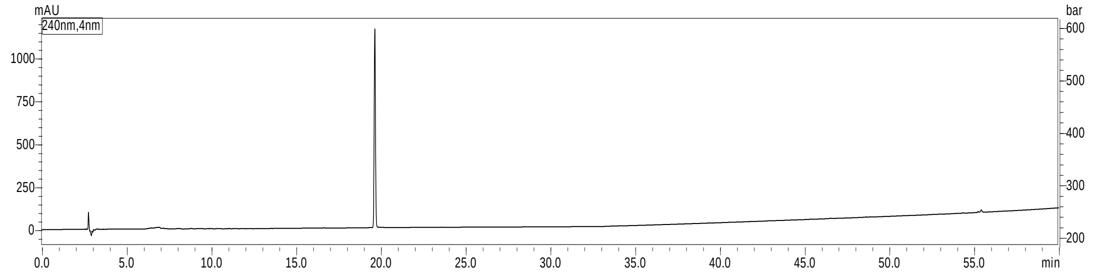

Figure S1. UV chromatogram of loganic acid (1) registered with HPLC-DAD at λ = 240 nm.

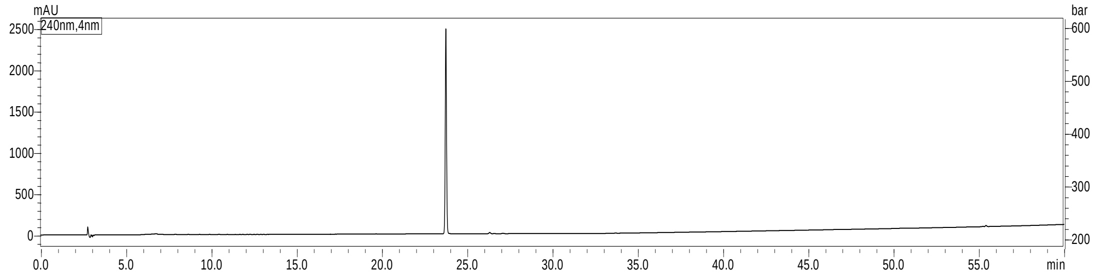

Figure S2. UV chromatogram of loganin (2) registered with HPLC-DAD at λ = 240 nm.

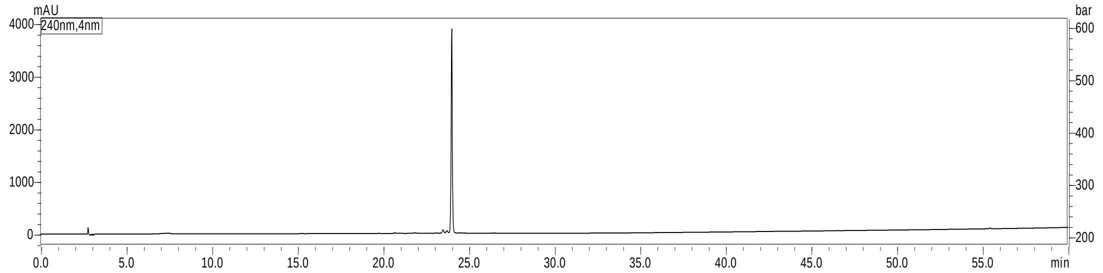

Figure S3. UV chromatogram of sweroside (3) registered with HPLC-DAD at λ = 240 nm.

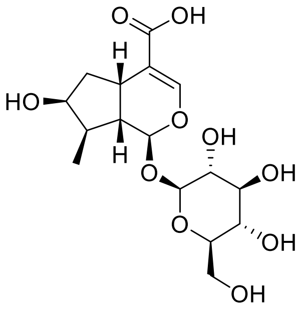

Loganic acid (1)

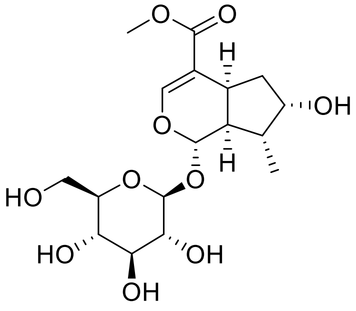

Loganin (2)

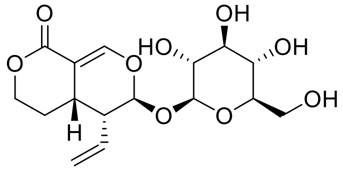

Sweroside (3)

Figure S4. The chemical structures of loganic acid (1), loganin (2), and sweroside (3).
